# Supplementary figures and images for: Transcriptomic analysis identifies genes and pathways related to myrmecophagy in the Malayan pangolin (Manis javanica)
Source: PeerJ. 2017 Dec 22;5:e4140. doi: 10.7717/peerj.4140 (PMC5742527; doi:10.7717/peerj.4140)

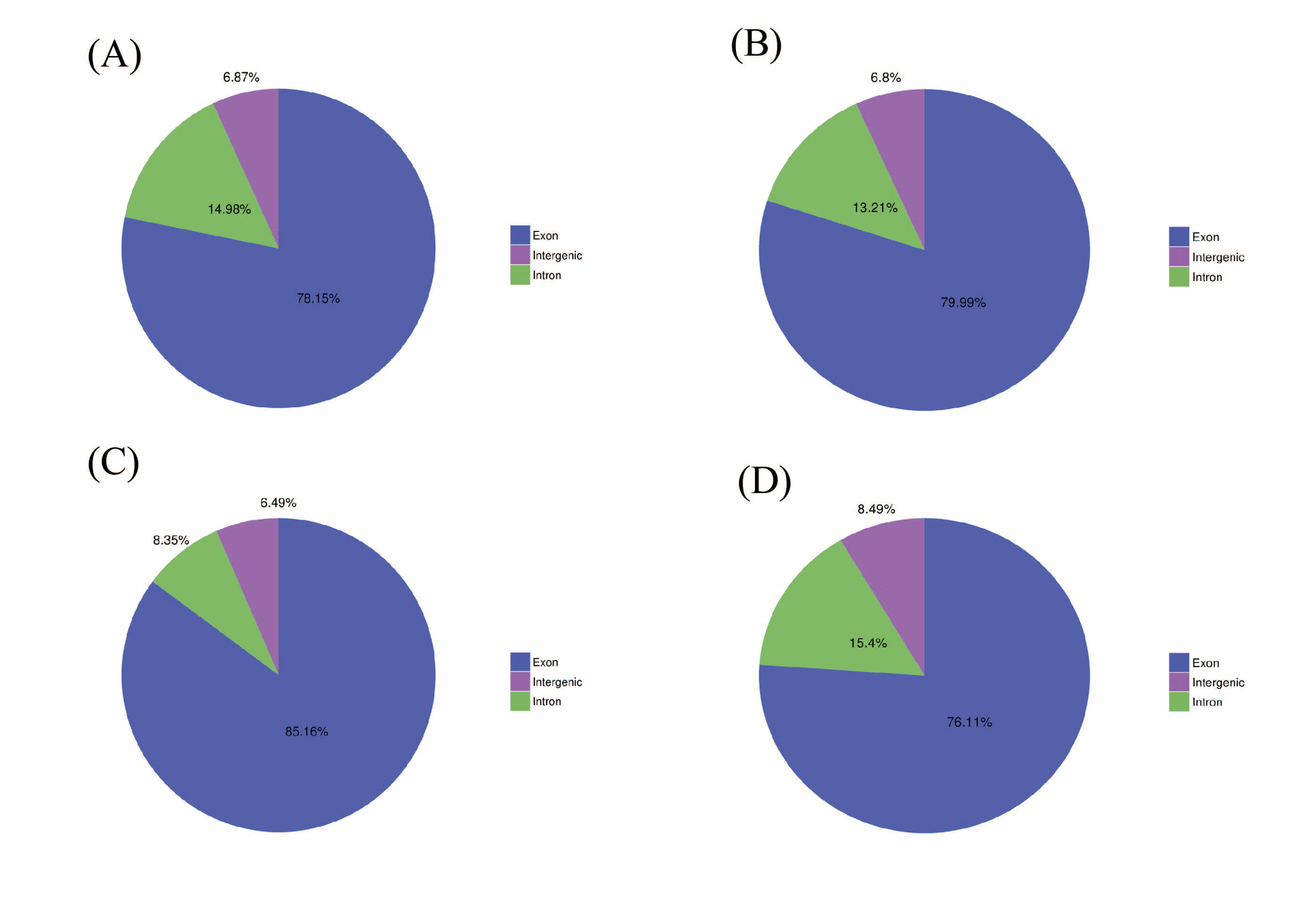

Supplement: Figure S1 [file peerj-05-4140-s008.png]

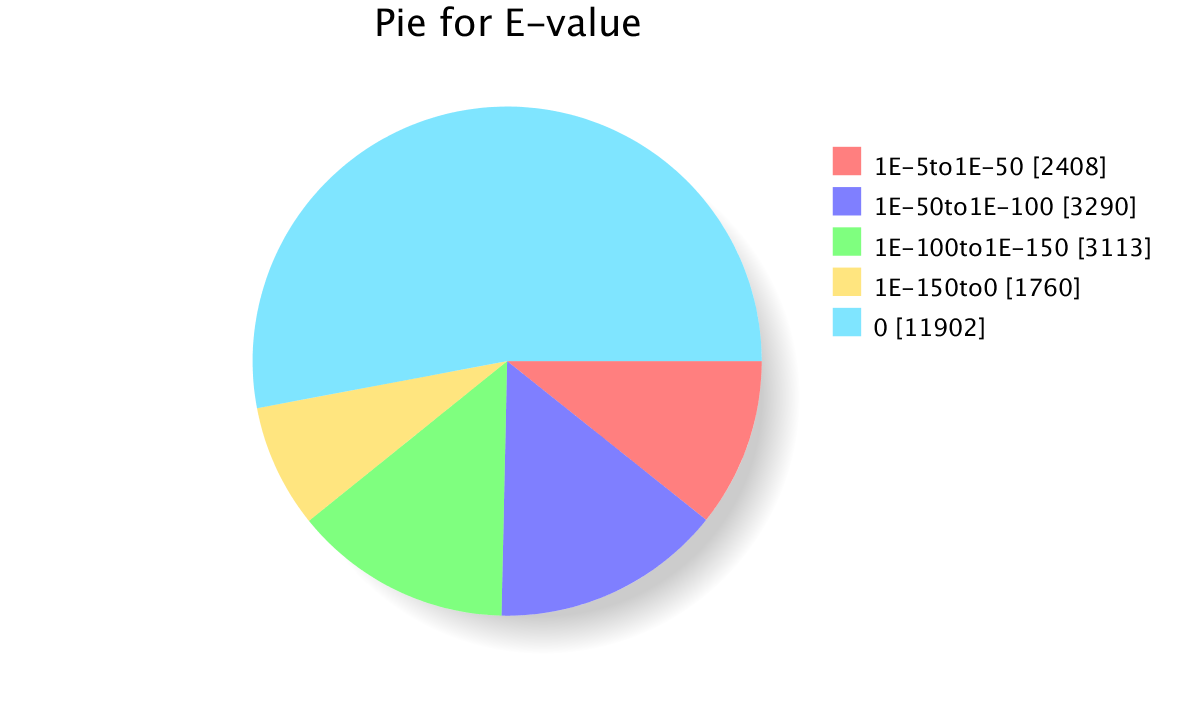

Supplement: Figure S2 [file peerj-05-4140-s009.png]

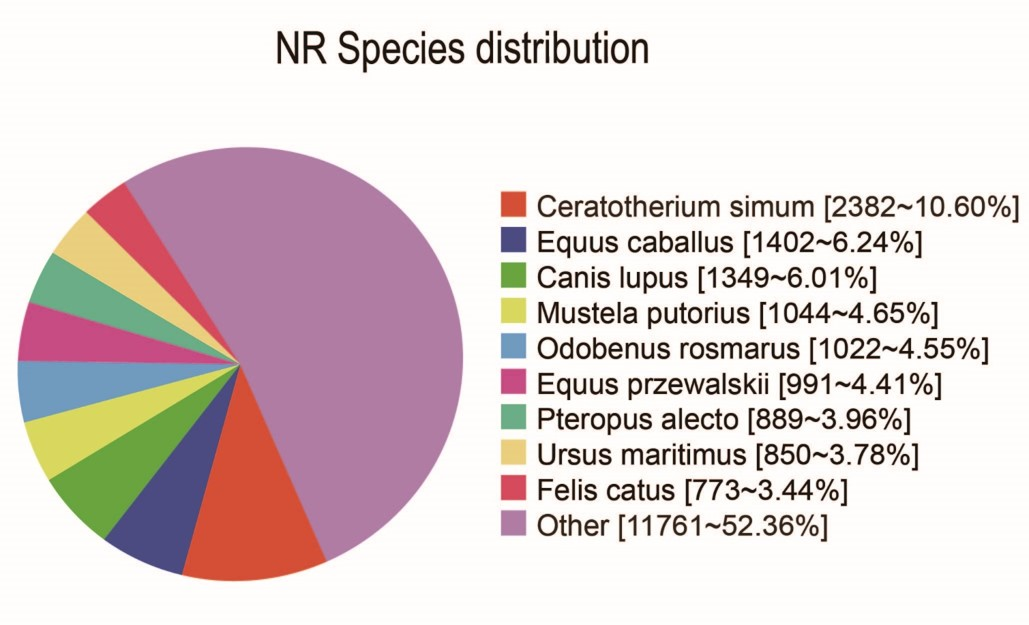

Supplement: Figure S3 [file peerj-05-4140-s010.png]

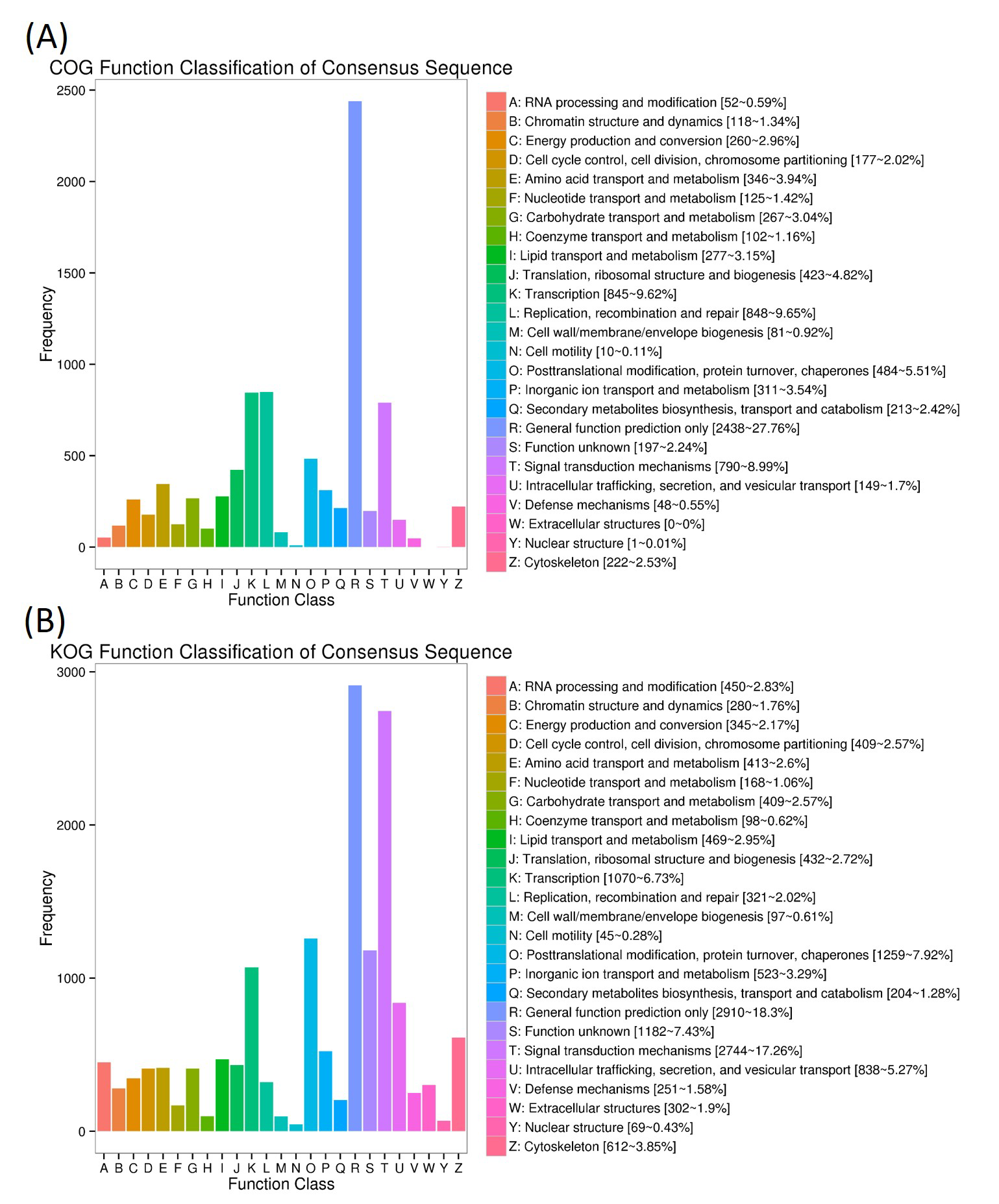

Supplement: Figure S4 — The x-axis shows the COG or KOG function classes, and the y-axis shows the number of transcripts in one class. The notation on the right shows the full names of the function classes. [file peerj-05-4140-s011.png]

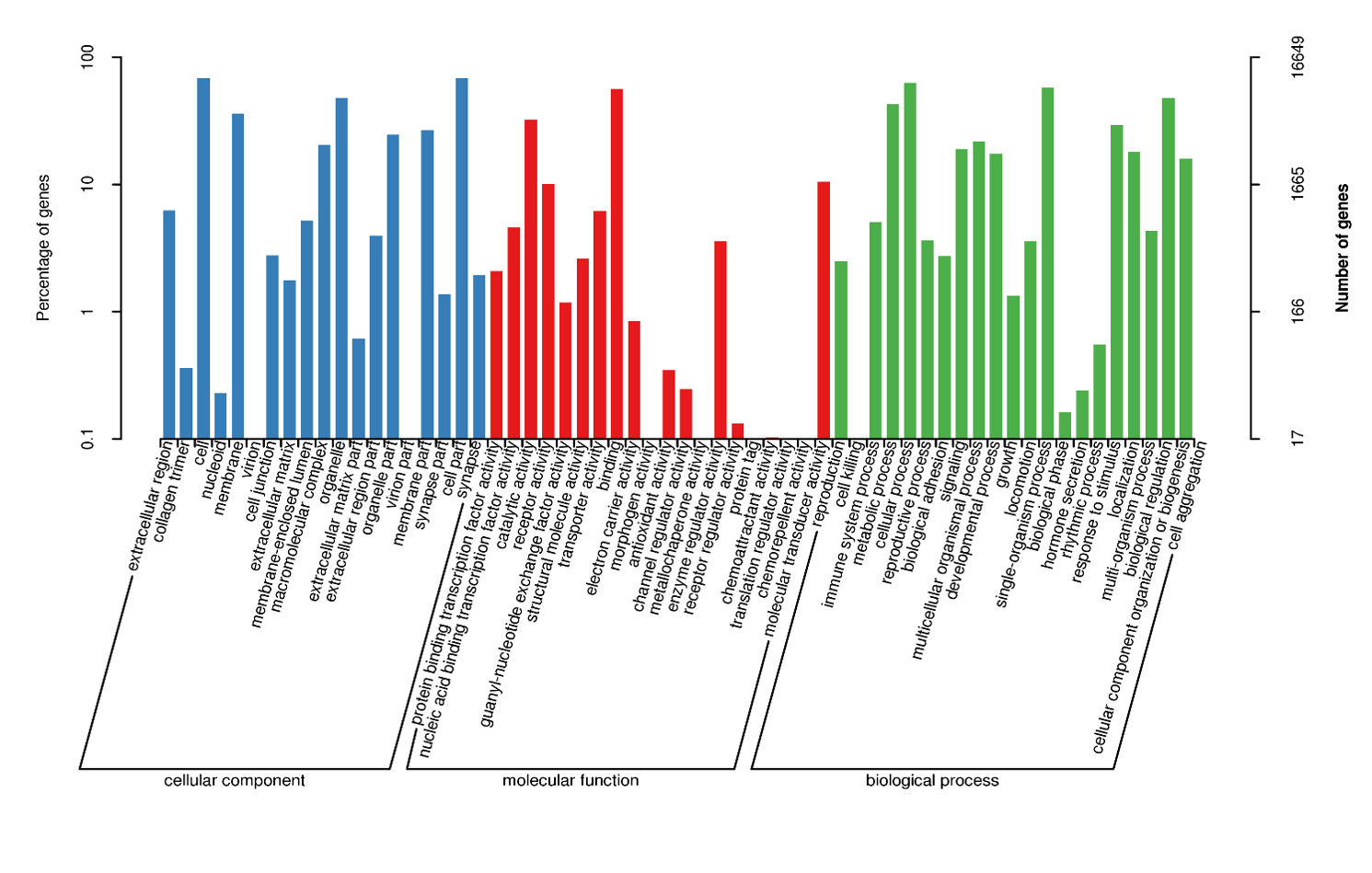

Supplement: Figure S5 — The x-axis shows GO function classes. The right side of the y-axis shows the number of transcripts with the GO function, and the left side shows the percentage. [file peerj-05-4140-s012.png]

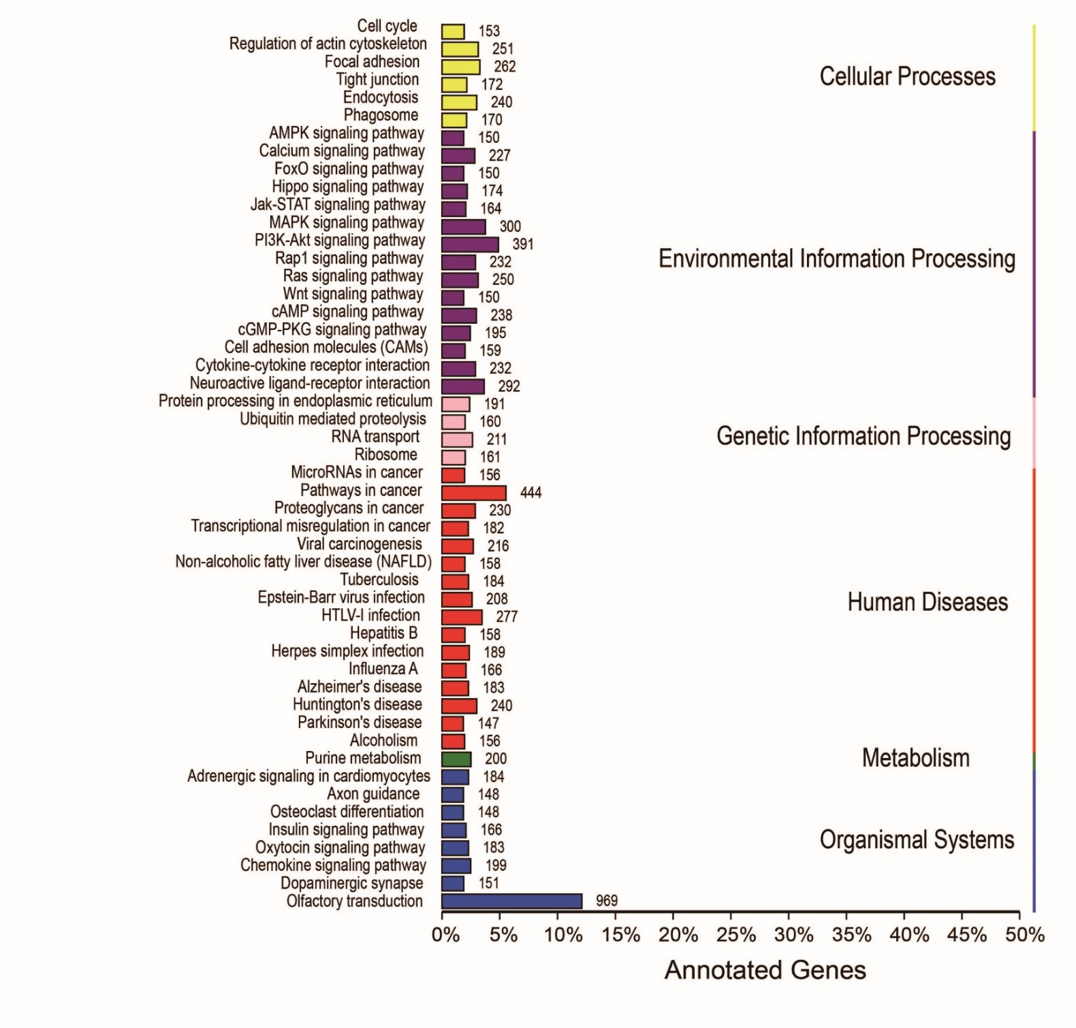

Supplement: Figure S6 [file peerj-05-4140-s013.png]

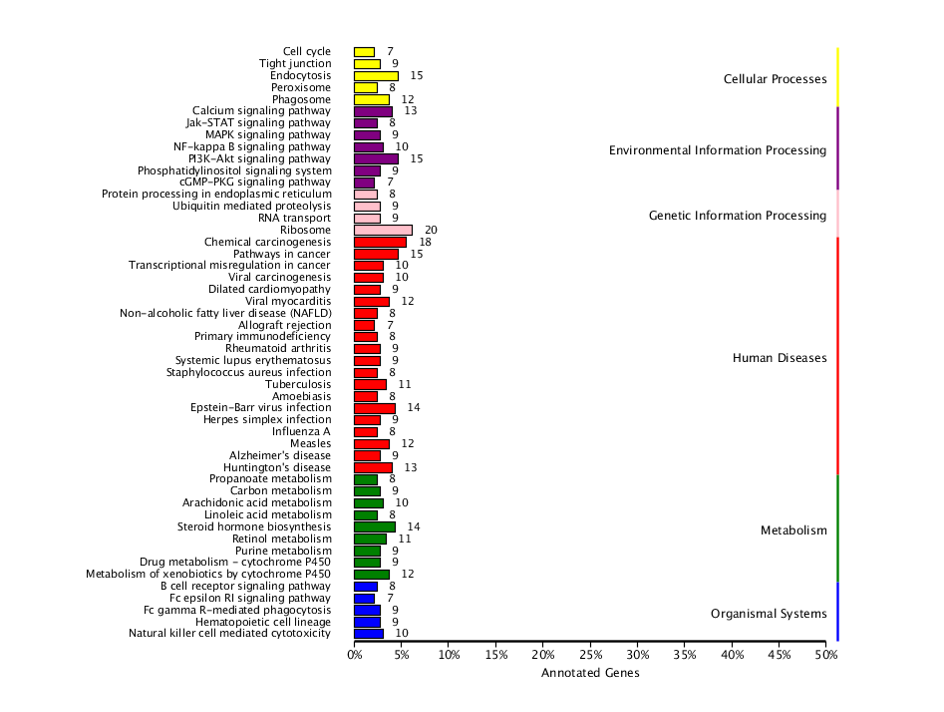

Supplement: Figure S7 — The x-axis shows the percentage of transcripts in one class, and the y-axis shows the KEGG function classes, the number of transcripts with the KEGG function class was shown above the column. [file peerj-05-4140-s014.png]

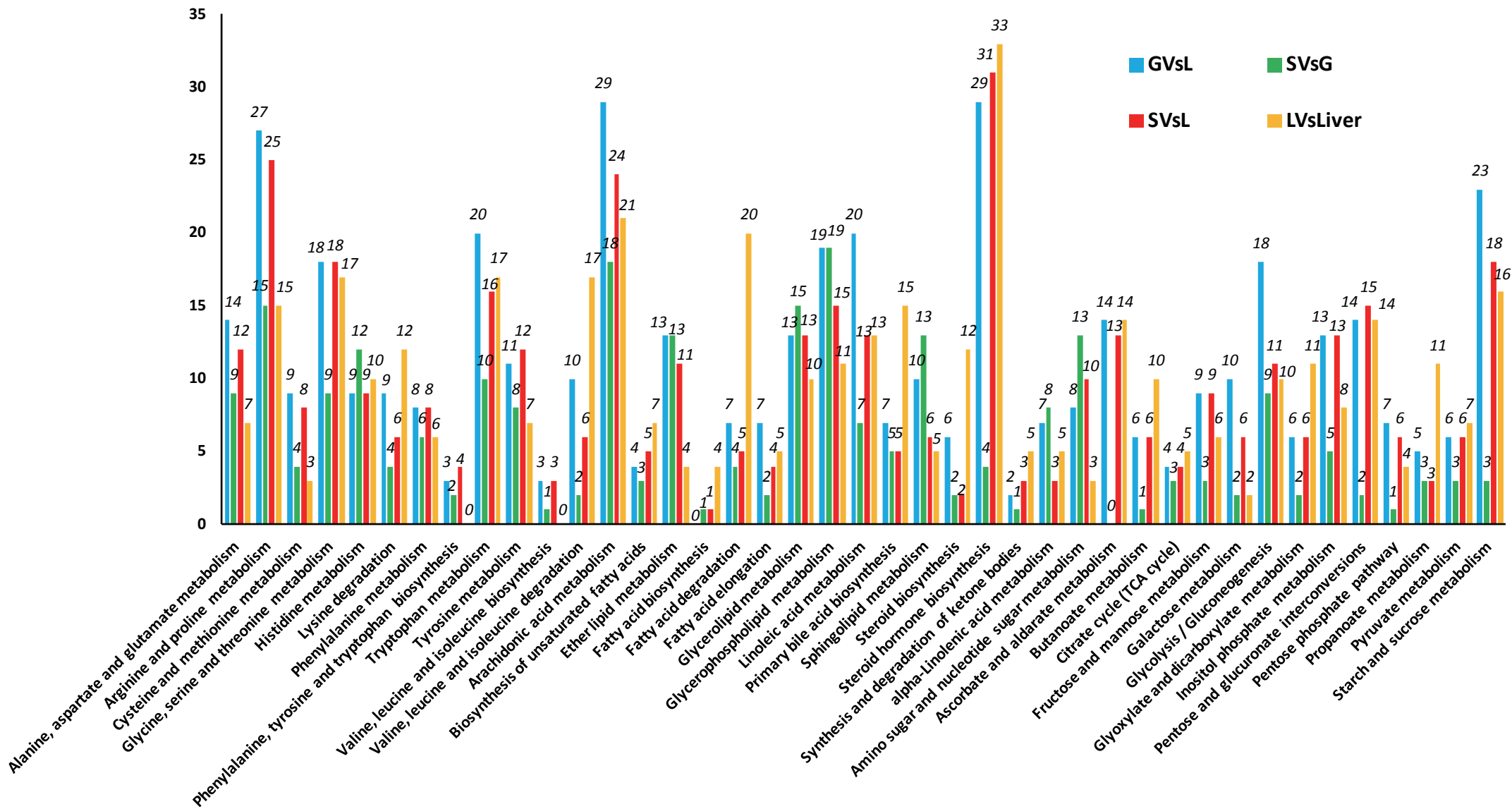

Supplement: Figure S8 — G, L, S and liver represent small intestine, liver, salivary glands and referred liver, respectively. [file peerj-05-4140-s015.pdf]
