# Supplementary material for: Transcriptomic analysis identifies genes and pathways related to myrmecophagy in the Malayan pangolin (Manis javanica)
Source: PeerJ. 2017 Dec 22;5:e4140. doi: 10.7717/peerj.4140 (PMC5742527; doi:10.7717/peerj.4140)
Supplement: Table S1 [file peerj-05-4140-s016.docx]

| #Anno_Database | Annotated_Number | 300<=length<1000 | length>=1000 |
| --- | --- | --- | --- |
| COG_Annotation | 6228 | 2022 | 4117 |
| GO_Annotation | 16648 | 6633 | 9519 |
| KEGG_Annotation | 13977 | 5475 | 8139 |
| KOG_Annotation | 14115 | 4971 | 8830 |
| Swissprot_Annotation | 17135 | 6908 | 9731 |
| TrEMBL_Annotation | 20964 | 8557 | 11827 |
| nr_Annotation | 22473 | 9156 | 12622 |
| All_Annotated | 22538 | 9198 | 12644 |
