# Supplementary material for: Transcriptomic analysis identifies genes and pathways related to myrmecophagy in the Malayan pangolin (Manis javanica)
Source: PeerJ. 2017 Dec 22;5:e4140. doi: 10.7717/peerj.4140 (PMC5742527; doi:10.7717/peerj.4140)
Supplement: Table S2 [file peerj-05-4140-s017.docx]

| #Anno_Database | Annotated_Number | 300<=length<1000 | length>=1000 |
| --- | --- | --- | --- |
| COG_Annotation | 145 | 56 | 75 |
| GO_Annotation | 662 | 279 | 302 |
| KEGG_Annotation | 619 | 280 | 276 |
| Swissprot_Annotation | 761 | 270 | 414 |
| nr_Annotation | 1446 | 553 | 780 |
| All_Annotated | 1459 | 559 | 787 |
