# Supplementary material for: Transcriptomic analysis identifies genes and pathways related to myrmecophagy in the Malayan pangolin (Manis javanica)
Source: PeerJ. 2017 Dec 22;5:e4140. doi: 10.7717/peerj.4140 (PMC5742527; doi:10.7717/peerj.4140)
Supplement: Table S3 [file peerj-05-4140-s018.docx]

| Sensory perception | Opsin | Rhodopsin kinase (GRK1), short-wave-sensitive opsin 1 (OPN1SW), long-wave-sensitive opsin 1 (OPN1LW), melanopsin (OPN4), phosphodiesterase 6D (PDE6D), retinal rod rhodopsin-sensitive cGMP 3&apos (PDE6G), retinal cone rhodopsin-sensitive cGMP 3&apos (PDE6H), rhodopsin (RHO) |
| --- | --- | --- |
|  | Taste | Taste receptor type 1 member 2 (TAS1R2), taste receptor type 1 member 3 (TAS1R3), taste receptor type 2 member 1 (TAS2R1), taste receptor type 2 member 4 (TAS2R4), taste receptor type 2 member 7 (TAS2R7), taste receptor type 2 member 10 (TAS2R10), taste receptor type 2 member 30 (TAS2R30), taste receptor type 2 member 38 (TAS2R38), taste receptor type 2 member 40 (TAS2R40) |
|  | Olfactor-y | Cyclic nucleotide-gated olfactory channel (CNGA2), olfactory receptor-like protein (DTMT), olfactory receptor (OR1A1, OR1D2, OR1E1, OR1E2, OR1E5, OR1G1, OR3A1, OR3A2, OR3A3), olfactory receptor-like protein (OLF1, OLF2, OLF3, OLF4) |
| Digestive  Enzyme  genes | Carbohy-drases | Amylo-alpha-1,6-glucosidase (AGL), pancreatic alpha-amylase (AMY2), acidic mammalian chitinase (CHIA), chitinase-3-like protein 1 (CHI3L1), chitinase domain-containing protein 1 (CHID1), lysosomal alpha-glucosidase (GAA), neutral alpha-glucosidase AB (GANAB), neutral alpha-glucosidase C (GANC), cytosolic beta-glucosidase (GBA3), cytosolic beta-glucosidase Beta-galactosidase (GLB1), beta-galactosidase-1-like protein (GLB1L), glucosidase 2 subunit beta (PRKCSH), isomaltase (SI) |
|  | Lipases | Monoacylglycerol lipase (ABHD6, ABHD12), bile salt-activated lipase (CEL), colipase (CLPS), phospholipase DDHD1, phosphatidylinositol-glycan-specific phospholipase D (GPLD1), group XV phospholipase A2, lysosomal acid lipase/cholesteryl ester hydrolase (LIPA), hepatic triacylglycerol lipase (LIPC), hormone-sensitive lipase (LIPE), gastric triacylglycerol lipase (LIPF), lipase member H (LIPH), lipase maturation factor 1 (LMF1), lipase maturation factor 2 (LMF2), lipoprotein lipase (LPL), lysophospholipase-like protein 1 (LYPLAL1), N-acyl-phosphatidylethanolamine-hydrolyzing phospholipase D (NAPEPLD), phospholipase A1 member A (PLA1A), phospholipase A2 (PLA2G1B), phospholipase A2, membrane associated (PLA2G2A), group 3 secretory phospholipase A2 (PLA2G3), lysophospholipase (PLA2G4A), soluble secretory phospholipase A2 receptor (PLA2R1), PLB1, phospholipase B-like 1 chain C (PLBD1), putative phospholipase B-like 2 (PLBD2), phospholipase D3 (PLD3), pancreatic triacylglycerol lipase (PNLIP), inactive pancreatic lipase-related protein 1 (PNLIPRP1), pancreatic lipase-related protein 2 (PNLIPRP2), patatin-like phospholipase domain-containing protein 2 (PNPLA2), calcium-independent phospholipase A2-gamma (PNPLA8) |
|  | Protease | Anionic trypsin, aminopeptidase N (ANPEP), cationic trypsin, chymotrypsin-like elastase family member 1 (CELA1), chymotrypsin A chain C (CTRB1), chymotrypsin-C (CTRC), dipeptidyl aminopeptidase-like protein 6 (DPP6), aspartyl aminopeptidase (DNPEP), glutamyl aminopeptidase (ENPEP), endoplasmic reticulum aminopeptidase 2 (ERAP2), cytosol aminopeptidase (LAP3), methionine aminopeptidase (METAP1, METAP2), probable aminopeptidase (NPEPL1), gastricsin (PGC), neurotrypsin (PRSS12), trypsin, probable Xaa-Pro aminopeptidase (XPNPEP1, XPNPEP2, XPNPEP3). |
| Transporte-rs | Sugar transport-er | Facilitated glucose transporter member (SLC2A1, SLC2A2, SLC2A3, SLC2A4, SLC2A5, SLC2A8, SLC2A9, SLC2A12), probable UDP-sugar transporter (SLC35A4, SLC35A5), sugar transporter (SLC50A1), UDP-N-acetyl glucosamine transporter (SLC35A3, SLC35B4, SLC35D2) |
|  | AA transport-er | Excitatory amino acid transporter (SLC1A1, SLC1A3, SLC1A6), neutral amino acid transporter (SLC1A4, SLC1A5, SLC7A8, SLC43A2, SLC6A15, SLC6A17, SLC6A19, SLC38A1, SLC38A2, SLC38A4, SLC38A5, SLC38A7, SLC38A10, SLC38A11), cationic amino acid transporter 2 (SLC7A2, SLC7A14), cystine/glutamate transporter (SLC7A11), mitochondrial basic amino acids transporter (SLC25A29) |
|  | Apolipo-protein | Truncated apolipoprotein A-I (APOA1), truncated apolipoprotein A-II (APOA2), apolipoprotein B (APOB), apolipoprotein A-IV, proapolipoprotein C-II (APOC2), apolipoprotein C-III (APOC3), apolipoprotein C-IV (APOC4), apolipoprotein D (APOD), apolipoprotein E (APOE), apolipoprotein M (APOM), apolipoprotein O (APOO) |
|  | Cationic/anion transport-er | H(+)/Cl(-) exchange transporter (CLCN3, CLCN5, CLCN7), molybdate-anion transporter (MFSD5), magnesium transporter (MAGT1, MMGT1, MRS2, NIPA2, NIPAL1), sodium-independent sulfate anion transporter, Electrogenic sodium bicarbonate cotransporter 1 (SLC4A4), sodium-dependent phosphate transporter (SLC20A1, SLC20A2), anion transporter (SLCO1C1, SLCO3A1, SLCO4C1) |
|  | Vitamin transport-er | Probable lysosomal cobalamin transporter (LMBRD1), proton-coupled folate transporter, sodium-dependent multivitamin transporter (SLC5A6), thiamine transporter (SLC19A3), mitochondrial folate transporter (SLC25A32), riboflavin transporter (SLC52A2, SLC52A3) |
|  | Cotransp-orter | Sodium/glucose cotransporter (SLC5A1, SLC5A4, SLC5A10), sodium/nucleoside cotransporter (SLC5A2, SLC28A1), sodium/myo-inositol cotransporter (SLC5A3) |
|  | Others | Sodium-dependent noradrenaline transporter (SLC6A2), sodium-dependent dopamine transporter (SLC6A3), sodium-dependent serotonin transporter (SLC6A4), sodium- and chloride-dependent transporter (SLC6A8, SLC6A9, SLC6A12, SLC6A13), ileal sodium/bile acid cotransporter (SLC10A2), monocarboxylate transporter (SLC5A12, SLC16A1, SLC16A9, SLC16A13), vesicular glutamate transporter (SLC17A6, SLC17A7), sulfate transporter (SLC26A2), equilibrative nucleoside transporter 3 (SLC29A3), choline transporter-like protein 2 (SLC44A2, SLC44A3, SLC44A4, SLC44A5), membrane-associated transporter protein (SLC45A2), thymic stromal cotransporter homolog (SLC46A2) |
