# Supplementary material for: Transcriptomic analysis identifies genes and pathways related to myrmecophagy in the Malayan pangolin (Manis javanica)
Source: PeerJ. 2017 Dec 22;5:e4140. doi: 10.7717/peerj.4140 (PMC5742527; doi:10.7717/peerj.4140)
Supplement: Table S4 [file peerj-05-4140-s019.docx]

| Tissue  Gene Name | Small intestine | Liver | Salivary glands | Referred liver |
| --- | --- | --- | --- | --- |
| Alanine, aspartate and glutamate metabolism | Glutamate decarboxylase 2 (GAD2) | 0 | 0 | 0 |
| Glycine, serine and threonine metabolism | 0 | Membrane primary amine oxidase (AOC3) | 0 | betaine--homocysteine S-methyltransferase 1 isoform X2 |
| Cysteine and methionine metabolism | Enolase-phosphatase | 0 | 0 | betaine--homocysteine S-methyltransferase 1 isoform X2 |
| Valine, leucine and isoleucine degradation | Aldehyde oxidase 2 (AOX2) | 0 | 0 | Manis_javanica_newGene_9458;Probable 3-hydroxymethyl-3-methylglutaryl-CoA lyase 2;hydroxymethylglutaryl-CoA synthase |
| Lysine degradation | 0 | 0 | 0 | Manis_javanica_newGene_9458 |
| Arginine and proline metabolism | Prolyl 4-hydroxylase subunit alpha-3 (P4HA3); Carnosine synthase 1 | 0 | 0 | ornithine carbamoyltransferase |
| Histidine metabolism | Carnosine synthase 1 | 0 | Retinal dehydrogenase 1 (ALDH1A1) | Manis_javanica_newGene_7661 |
| Tyrosine metabolism | Aldehyde oxidase 2 (AOX2) | L-dopachrome tautomerase;  Membrane primary amine oxidase (AOC3) | Tyrosine 3-monooxygenase;Retinal dehydrogenase 1 (ALDH1A1) | 0 |
| Phenylalanine metabolism | 0 | Membrane primary amine oxidase (AOC3) | Retinal dehydrogenase 1 (ALDH1A1) | Manis_javanica_newGene_13705;glycine N-phenylacetyltransferase-like |
| Tryptophan metabolism | Aldehyde oxidase 2 (AOX2);  Indoleamine 2,3-dioxygenase | 0 | 0 | Manis_javanica_newGene_9458;cytochrome P450 1A1-like;Cytochrome P450 |
| Fatty acid elongation | Elongation of very long chain fatty acids protein 7 (ELOVL7) | 0 | 0 | 0 |
| Fatty acid degradation | Carnitine O-palmitoyltransferase 1, liver isoform (CPT1A) | 0 | 0 | Manis_javanica_newGene_9458;Carbonyl reductase family member 4 (CBR4) |
| Synthesis and degradation of ketone bodies | 0 | 0 | 0 | Manis_javanica_newGene_9458;Probable 3-hydroxymethyl-3-methylglutaryl-CoA lyase 2;hydroxymethylglutaryl-CoA synthase |
| Steroid biosynthesis | regulator of G-protein signaling 22 | 0 | 0 | Sterol O-acyltransferase 2 (SOAT2);Cytochrome P450 2J1 (CYP2J1) |
| Primary bile acid biosynthesis | 0 | 0 | 0 | Manis_javanica_newGene_8788;Cholesterol 7-alpha-monooxygenase (CYP7A1);24-hydroxycholesterol 7-alpha-hydroxylase-like isoform X4 |
| Steroid hormone biosynthesis | 0 | Cytochrome P450c17 | 0 | 0 |
| Glycerolipid metabolism | 1-acyl-sn-glycerol-3-phosphate acyltransferase delta (AGPAT4) | glycerol-3-phosphate acyltransferase 2 | Inactive pancreatic lipase-related protein 1 (PNLIPRP1);Diacylglycerol kinase alpha (DGKA) | 0 |
| Glycerophospholipid metabolism | Phospholipase A2, membrane associated (PLA2G2A); 1-acyl-sn-glycerol-3-phosphate acyltransferase delta (AGPAT4);gene22988;cytosolic phospholipase A2 delta;group 3 secretory phospholipase A2;Phospholipase A2 (PLA2G1B) | gene20840; glycerol-3-phosphate acyltransferase 2 | Diacylglycerol kinase alpha (DGKA) | Phospholipase D3 (PLD3) |
| Ether lipid metabolism | Phospholipase A2, membrane associated (PLA2G2A);gene17228; Lysophospholipase (PLA2G4A);cytosolic phospholipase A2 delta;group 3 secretory phospholipase A2;Phospholipase A2 (PLA2G1B) | Lysophospholipase (PLA2G4A) | 0 | Phospholipase D3 (PLD3) |
| Sphingolipid metabolism | Voltage-dependent calcium channel gamma-3 subunit (CACNG3);Sialidase-3 (NEU3);sphingosine-1-phosphate phosphatase 2 | 0 | 0 | Manis_javanica_newGene_13789;  alkaline ceramidase 3 isoform X1 |
| Arachidonic acid metabolism | Phospholipase A2, membrane associated (PLA2G2A);gene21148; Lysophospholipase (PLA2G4A);cytosolic phospholipase A2 delta;group 3 secretory phospholipase A2;Phospholipase A2 (PLA2G1B) | Lysophospholipase (PLA2G4A) | Diacylglycerol kinase alpha (DGKA) | Manis_javanica_newGene_11007;  Manis_javanica_newGene_12165;  Manis_javanica_newGene_12624;  Manis_javanica_newGene_8960;  Cytochrome P450 4A7 (CYP4A7);Arachidonate 15-lipoxygenase (ALOX15);Cytochrome P450 4A6 (CYP4A6);Carbonyl reductase [NADPH] 1 (CBR1) |
| Linoleic acid metabolism | Phospholipase A2, membrane associated (PLA2G2A); Lysophospholipase (PLA2G4A);cytosolic phospholipase A2 delta;group 3 secretory phospholipase A2;Phospholipase A2 (PLA2G1B) | Lysophospholipase (PLA2G4A) | 0 | Cytochrome P450 3A12 (CYP3A12);Cytochrome P450 3A8 (CYP3A8) |
| alpha-Linolenic acid metabolism | Phospholipase A2, membrane associated (PLA2G2A); Lysophospholipase (PLA2G4A);cytosolic phospholipase A2 delta;group 3 secretory phospholipase A2;Phospholipase A2 (PLA2G1B) | Lysophospholipase (PLA2G4A) | 0 | 0 |
| Biosynthesis of unsaturated fatty acids | 0 | 0 | Stearoyl-CoA desaturase 5 (SCD5) | 0 |
| Starch and sucrose metabolism | Nucleotide pyrophosphatase (ENPP3) | 0 | 0 | 0 |
| Glycolysis / Gluconeogenesis | 0 | 0 | Retinal dehydrogenase 1 (ALDH1A1) | Manis_javanica_newGene_5972 |
| Citrate cycle (TCA cycle) | 0 | 0 | 0 | Manis_javanica_newGene_5972 |
| Pentose and glucuronate interconversions | 0 | 0 | 0 | Manis_javanica_newGene_8627;UDP-glucuronosyltransferase 2B31 (UGT2B31) |
| Ascorbate and aldarate metabolism | 0 | 0 | 0 | Manis_javanica_newGene_8627;UDP-glucuronosyltransferase 2B31 (UGT2B31);Inositol oxygenase (MIOX) |
| Pyruvate metabolism | 0 | 0 | 0 | Manis_javanica_newGene_5972;Manis_javanica_newGene_9458;acyl-coenzyme A thioesterase 12 |
| Amino sugar and nucleotide sugar metabolism | 0 | 0 | 0 | NADH-cytochrome b5 reductase 1 (CYB5R1) |
| Pyruvate metabolism | 0 | 0 | 0 | Manis_javanica_newGene_5972;Manis_javanica_newGene_9458;acyl-coenzyme A thioesterase 12 |
| Glyoxylate and dicarboxylate metabolism | 0 | 0 | 0 | Manis_javanica_newGene_9458;Hydroxyacid oxidase 2 (HAO2) |
| Propanoate metabolism | 0 | 0 | 0 | Manis_javanica_newGene_9458 |
| Proximal tubule bicarbonate reclamation | 0 | 0 | Sodium/potassium-transporting ATPase subunit gamma (FXYD2) | 0 |
